# Evaluation of Amebicidal and Cysticidal Activities of Antifungal Drug Isavuconazonium Sulfate against *Acanthamoeba* T4 Strains

**DOI:** 10.3390/ph14121294

**Published:** 2021-12-11

**Authors:** Brian Shing, Mina Balen, Anjan Debnath

**Affiliations:** 1 Biomedical Sciences Graduate Division, University of California San Diego, 9500 Gilman Drive, MC 0685, La Jolla, CA 92093-0756, USA; bjshing@health.ucsd.edu; 2 Center for Discovery and Innovation in Parasitic Diseases, Skaggs School of Pharmacy and Pharmaceutical Sciences, University of California San Diego, 9500 Gilman Drive, MC 0756, La Jolla, CA 92093-0756, USA; mbalen@ucsd.edu; 3 Division of Biological Sciences, University of California San Diego, 9500 Gilman Drive, MC 0346, La Jolla, CA 92093-0756, USA

**Keywords:** *Acanthamoeba*, free-living ameba, *Acanthamoeba* keratitis, isavuconazonium sulfate, cyst, drug

## Abstract

*Acanthamoeba* species of amebae are often associated with *Acanthamoeba* keratitis, a severe corneal infection. Isavuconazonium sulfate is an FDA-approved drug for the treatment of invasive aspergillosis and mucormycosis. This prodrug is metabolized into the active isavuconazole moiety. Isavuconazole was previously identified to have amebicidal and cysticidal activity against *Acanthamoeba* T4 strains, but the activity of its prodrug, isavuconazonium sulfate, against trophozoites and cysts remains unknown. Since it is not known if isavuconazonium can be metabolized into isavuconazole in the human eye, we evaluated the activities of isavuconazonium sulfate against trophozoites and cysts of three T4 genotype strains of *Acanthamoeba.* Isavuconazonium displayed amebicidal activity at nanomolar concentrations as low as 1.4 nM and prevented excystation of cysts at concentrations as low as 136 μM. We also investigated the cysticidal activity of isavuconazonium sulfate in combination with a currently used amebicidal drug polyhexamethylene biguanide (PHMB). Although combination of isavuconazonium with PHMB did not elicit an obvious synergistic cysticidal activity, the combination did not cause an antagonistic effect on the cysts of *Acanthamoeba* T4 strains. Collectively, these findings suggest isavuconazonium retains potency against *Acanthamoeba* T4 strains and could be adapted for *Acanthamoeba* keratitis treatment.

## 1. Introduction

*Acanthamoeba castellanii* is a causative agent of *Acanthamoeba* keratitis (AK). It is a serious infection of the eye that causes inflammation in the cornea and can result in permanent visual impairment or blindness. *Acanthamoeba* is common in nature and can be found in soil, air and water, including insufficiently chlorinated pools, hot tubs, tap and shower water. In unfavorable environments, the ameboid form of the organism called a ‘trophozoite’ transforms into a drug-resistant double-walled cyst. Cyst resistance to therapeutic agents, and recurrence of infection due to *Acanthamoeba* excystment, remain challenges for disease prevention and cures. Infection recurrence occurs in approximately 10% of cases [1], due possibly to excystment. No single drug has yet been shown effective at therapeutic concentrations against both the trophozoite and cyst stages of *Acanthamoeba*. Current treatment of AK involves an aggressive disinfectant chlorhexidine, in combination with diamidines, polyhexamethylene biguanide (PHMB) and neomycin. Combination therapies have proven more successful than monotherapies [2,3,4]. The most aggressive and severe cases of AK require corneal grafts or surgical removal of the eye [5]. Despite advances in combination therapies and surgery, the resistance of cysts to therapeutic agents poses challenges that are yet to be addressed [6]. Therefore, discovering and identifying therapeutics that are effective against both stages of the parasite would be critical to reducing AK recurrence and improving existing therapies.

Earlier, we identified isavuconazole as amebicidal and cysticidal [7], but clinically the prodrug isavuconazonium sulfate is administered orally or intravenously for the treatment of fungal infections. Isavuconazonium sulfate is metabolized by plasma esterase enzymes, specifically butyrylcholinesterase, into isavuconazole [8]. However, it is unknown if isavuconazonium can be metabolized into isavuconazole in the human eye. As such, we evaluated the activity of isavuconazonium sulfate activity against the trophozoites and cysts of three separate T4 genotype strains of *Acanthamoeba*.

## 2. Results

### 2.1. Determination of Amebicidal Activity

**Figure 1:**
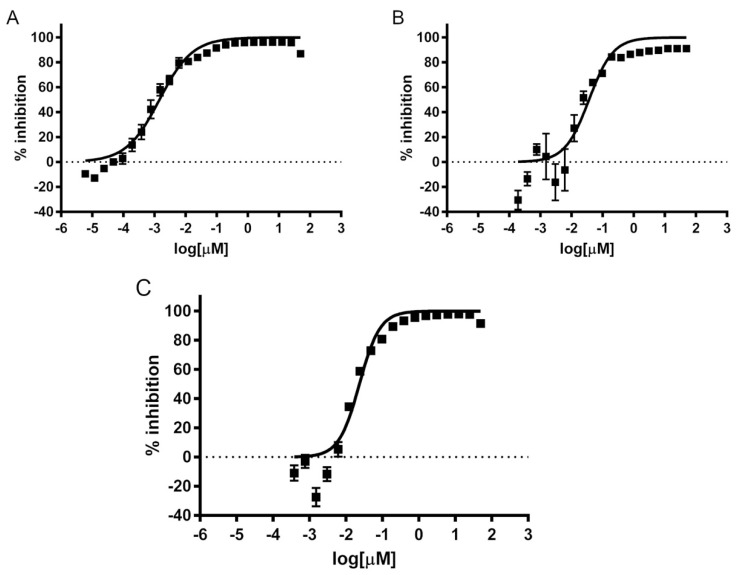
Concentration—dependent inhibition of growth of *Acanthamoeba* trophozoites by isavuconazonium sulfate. Different concentrations of isavuconazonium were tested in triplicate for activity against trophozoites of *Acanthamoeba* T4 strains. The data points represent mean percentage growth inhibition of (**A**) Ma strain, (**B**) CDC:V240, and (**C**) MEEI 0184 of different concentrations of isavuconazonium. EC_50_ curves were generated from mean values of percentage growth inhibition of isavuconazonium against *Acanthamoeba*.

The amebicidal activity of isavuconazonium sulfate was tested against three T4 genotype strains of *Acanthamoeba* (strains Ma, CDC:V240, and MEEI 0184). The trophozoites were exposed to serial dilutions of isavuconazonium sulfate with final concentrations ranging from 50 µM to 0.006 nM. All three strains of *Acanthamoeba* trophozoite appeared to be highly susceptible to isavuconazonium sulfate. Isavuconazonium displayed an EC_50_ of 0.001 µM against *Acanthamoeba* strain Ma (Figure 1A), which was about 1700- to 5000-fold more potent than the current standards of care chlorhexidine and PHMB. The EC_50_ of isavuconazonium against *Acanthamoeba* strain CDC:V240 was 0.037 µM (Figure 1B), which was about 30- to 300-fold more potent than chlorhexidine and PHMB, respectively. Isavuconazonium exhibited an EC_50_ of 0.024 µM against clinical strain MEEI 0184 (Figure 1C). This EC_50_ was about 1.5-fold better than the EC_50_ demonstrated against the CDC:V240 strain (Table 1). Overall, these nanomolar potencies demonstrate that isavuconazonium retains its potency against trophozoites of *Acanthamoeba* T4 strains.

### 2.2. Determination of Cysticidal Activity

*Acanthamoeba* T4 cysts are also clinically relevant, as they are often more difficult to treat than trophozoites and require higher concentrations of antimicrobial compounds for efficacy. In order to determine the activity of isavuconazonium sulfate against cysts of *Acanthamoeba* T4 strains, cysts from all three strains were tested against higher concentrations of isavuconazonium sulfate than for evaluating trophozoites. Final concentrations of isavuconazonium ranged from 200 μM to 100 μM in increments of 10 μM, and a treatment was considered to be cysticidal if there was no evidence of trophozoite proliferation or excystation by day 7, which is a commonly used end point for cysticidal assays [9,10,11,12,13].

**Figure 2:**
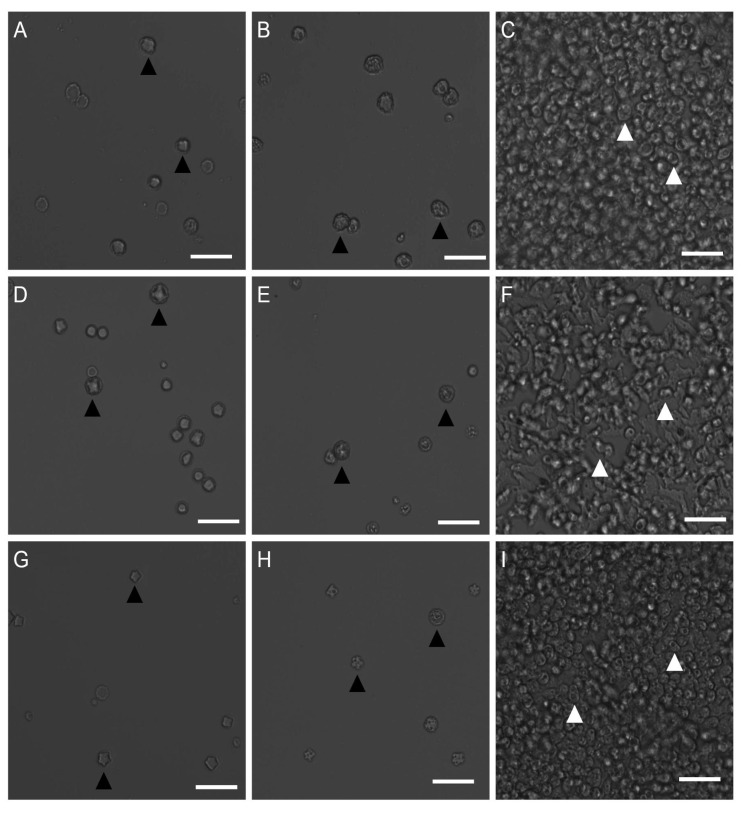
Effect of isavuconazonium sulfate on the morphology of *Acanthamoeba* Ma, CDC:V240 and MEEI 0184 cysts. Ma cysts were treated with (**A**) 170 μM isavuconazonium, (**B**) 461.85 μM PHMB, (**C**) 0.5% (*v*/*v*) DMSO. CDC:V240 strain cysts were treated with (**D**) 140 μM isavuconazonium, (**E**) 461.85 μM PHMB, (**F**) 0.5% (*v*/*v*) DMSO. MEEI 0184 strain cysts were treated with (**G**) 190 μM isavuconazonium, (**H**) 461.85 μM PHMB, (**I**) 0.5% (*v*/*v*) DMSO. Morphology and excystation of *Acanthamoeba* T4 cysts after 7 days of incubation in PYG growth media are displayed. Black arrowheads: cysts. White arrowheads: trophozoites. Magnification: 200×. Scale bar: 50 μm.

Acanthamoeba T4 cysts were treated with isavuconazonium and allowed to recover in PYG growth media for 7 days and evaluated for cysticidal activity. Isavuconazonium displayed cysticidal activity against all three tested strains, as no excystation was observed at day 7 (Figure 2A,D,G). Isavuconazonium exhibited an average minimum cysticidal concentration (MCC) of 167.1 ± 23.6 μM against *Acanthamoeba* Ma strain. *Acanthamoeba* strain CDC:V240 had an average MCC of 136.0 ± 11.4 µM. *Acanthamoeba* strain MEEI 0184 displayed an average MCC of 187.5 ± 5.0 µM.

### 2.3. Effect of Combination of Isavuconazonium and PHMB on Cysts

While isavuconazonium has low nanomolar potency against trophozoites, it appears to display cysticidal activity only at high micromolar concentrations. Since cysts require higher isavuconazonium concentrations to prevent excystation, we wanted to evaluate if isavuconazonium in combination with other currently used drugs can display synergy to reduce the isavuconazonium concentration required to treat cysts.

**Figure 3:**
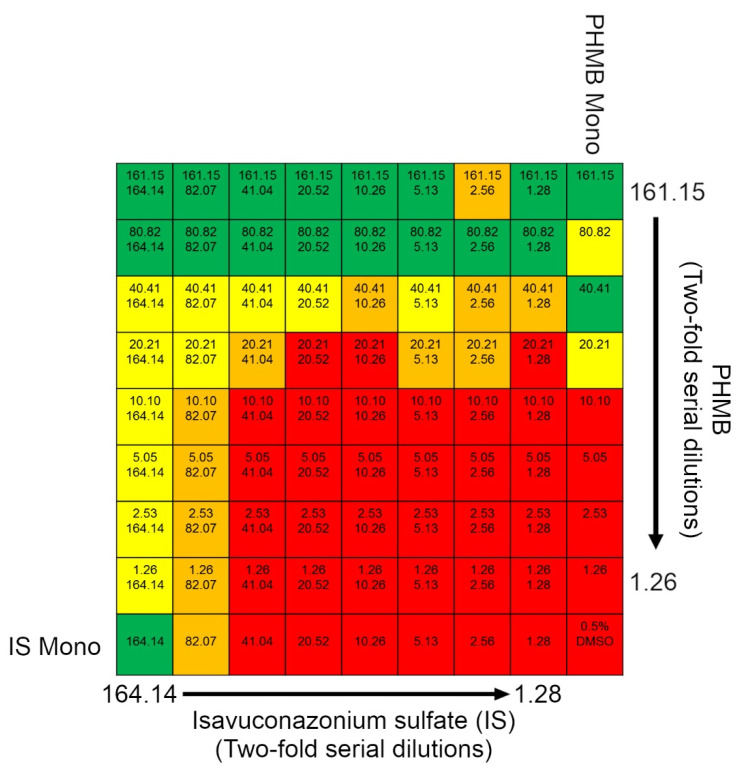
Isavuconazonium-PHMB combination excystation heatmap. Heatmap displaying qualitative scoring of excystation for different isavuconazonium sulfate (IS)–PHMB combination treatments. Top number in grids represents PHMB concentration in μM, bottom number in grids represents isavuconazonium sulfate concentration in μM. Green: 0% trophozoite plate coverage; yellow: 0–1% trophozoite plate coverage; orange: 50–80% trophozoite plate coverage; red: 100% trophozoite plate coverage.

**Figure 4:**
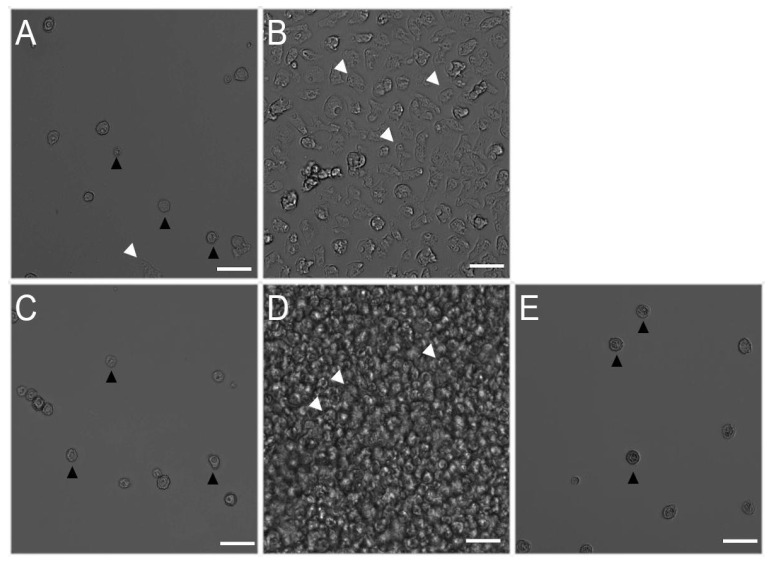
Effect of combination of isavuconazonium sulfate and PHMB on the morphology of *Acanthamoeba* Ma cysts. Ma cysts were treated with (**A**) 20.52 μM isavuconazonium and 40.41 μM PHMB, (**B**) 20.52 μM isavuconazonium monotherapy, (**C**) 40.41 μM PHMB monotherapy, (**D**) 0.5% (*v*/*v*) DMSO, (**E**) 461.85 μM PHMB. Morphology and excystation of *Acanthamoeba* cysts were evaluated after 7 days of incubation in PYG growth media. Black arrowheads: cysts. White arrowheads: trophozoites. Magnification: 200×. Scale bar: 50 μm.

Isavuconazonium was combined with PHMB and qualitatively assessed for excystation after 7 days of incubation in growth media (Figure 3). Isavuconazonium displayed an MCC of 167.1 μM. Combined with 40.41 μM PHMB, isavuconazonium was able to be lowered to 20.52 μM and still have minimal excystation (Figure 4A). Monotherapy of 20.52 μM isavuconazonium was confluent with trophozoites by day 7 (Figure 4B). Monotherapy of 40.41 μM PHMB had minimal to no excystation (Figure 4C). Since the combination of 20.52 µM of isavuconazonium with 40.41 µM of PHMB elicited a similar effect to what was caused by 40.41 µM of PHMB alone, it is apparent that the combination of two compounds did not have a synergistic effect able to suppress excystation or kill cysts. It was clear that the combination of these two compounds at this concentration did not cause an antagonistic effect on the cysts of Ma strain of *Acanthamoeba*.

## 3. Discussion

Isavuconazole has previously been evaluated and demonstrated potent amebicidal and cysticidal activity [7]. Since isavuconazole is typically administered as the prodrug isavuconazonium sulfate, there is a possibility that it may not be metabolized in the human eye and that *Acanthamoeba* T4 strains would not be susceptible to isavuconazole. Previously, isavuconazonium was identified as effective against *Acanthamoeba* T4 trophozoites [14], but its effect was not investigated against multiple strains and, more importantly, on the cysts of *Acanthamoeba* T4 strains. In this work, we evaluated the amebicidal and cysticidal activity of isavuconazonium against multiple strains of *Acanthamoeba* T4, and also explored the possibility of combining isavuconazonium with PHMB against the cysts of *Acanthamoeba*.

Isavuconazonium had mean EC_50_ values against trophozoites ranging from 0.001 µM (strain Ma) to 0.037 µM (strain CDC:V240) (Table 1). Our reported values are lower than those reported by Rice et al. against *Acanthamoeba* strain Ma (EC_50_ of 0.09 ± 0.02 µM [14]. This could be due to the differences in experimental conditions. We also previously evaluated the active drug isavuconazole against trophozoites and reported an EC_50_ of <0.001 µM (strain CDC:V240), 0.005 µM (strain Ma), and 0.026 µM (strain MEEI 0184) [7]. The EC_50_ values of isavuconazonium for strains Ma and MEEI 0184 are comparable to those previously reported values for isavuconazole. Interestingly, the EC_50_ of isavuconazonium against CDC:V240 was approximately 40× higher than that of the previously reported isavuconazole EC_50_ value. Taken together, this suggests Acanthamoeba T4 trophozoites are still susceptible to the prodrug isavuconazonium.

To our knowledge, this is the first reported evaluation of the cysticidal activity of isavuconazonium sulfate against *Acanthamoeba* T4 strains. We previously reported that isavuconazole, the active form of isavuconazonium, displayed cysticidal activity against *Acanthamoeba* Ma at 70 μM [7]. In this work, we determined the MCC of isavuconazonium against various *Acanthamoeba* T4 strains. The MCC of isavuconazonium varies from 1.9× (CDC:V240) to 2.6× (MEEI 0184) higher than previously reported about isavuconazole [7]. Since isavuconazonium is a prodrug that must be metabolized to active isavuconazole, it is possible that higher concentrations of the prodrug are required as not all of the isavuconazonium is metabolized into isavuconazole.

AK therapies frequently rely on chlorhexidine or PHMB as monotherapy, or in combination with propamidine isethionate and hexamidine. Commonly used combinations include PHMB with propamidine, chlorhexidine with propamidine, chlorhexidine with PHMB, and PHMB with propamidine and neomycin [15]. Since combination therapies were found to be more successful than monotherapies, we investigated the effect of the combination of isavuconazonium and PHMB on cysts of an *Acanthamoeba* T4 strain. In spite of the challenges associated with the excystation-based cysticidal assay that depends on a “cysticidal-or-not” readout rather than percentage inhibition [16], we identified that the combination of isavuconazonium and PHMB did not cause antagonistic or synergistic cysticidal effects on the cysts of the Ma strain of *Acanthamoeba*. Future studies will require confirmation of the effect of the combination of isavuconazonium and PHMB on trophozoites and cysts of different strains. Whether isavuconazonium can be combined with other commonly used drugs will require further investigation.

Isavuconazonium sulfate is FDA-approved for the treatment of invasive aspergillosis and mucormycosis [17]. In terms of fungal infections, isavuconazonium sulfate inhibits lanosterol 14α-demethylase [8], which prevents the biosynthesis of ergosterol and results in its eventual depletion. It is available in both oral and intravenous formulations and, following administration, it is rapidly cleaved to the active isavuconazole. The tissue distribution of isavuconazole was evaluated in animals after oral and intravenous administrations of isavuconazonium sulfate, and a low concentration of isavuconazole was detected in the eye lens [17,18]. This low concentration of isavuconazole may not be sufficient to kill *Acanthamoeba* T4 cysts if the drug is administered orally or intravenously. The preferable route of administration of drugs for the treatment of AK is topical, but the distribution of isavuconazole in the eye has not been evaluated when administered topically. Therefore, it was important to determine the effect of the prodrug isavuconazonium sulfate in case topical administration of prodrug isavuconazonium sulfate does not lead to the formation of active isavuconazole in human eyes. The potent amebicidal and cysticidal activities of the prodrug isavuconazonium sulfate against multiple T4 strains of *Acanthamoeba* provide confidence that the FDA-approved isavuconazonium sulfate is a promising lead for the treatment of AK.

## 4. Materials and Methods

### 4.1. Cell Culture

*Acanthamoeba* strains Ma (American Type Culture Collection #50370, Manassas, USA), CDC:V240 (CDC, Atlanta, VA, USA), and MEEI 0184 (Tufts University, Medford, OR, USA), belonging to T4 genotype, were cultured as described by Shing et al. [7]. Trophozoites were cultured at 28 °C and 5% CO_2_ in peptone yeast glucose (PYG) medium supplemented with 100 U/mL penicillin and 100 μg/mL streptomycin.

### 4.2. Determination of Amebicidal Activity

Stock 10 mM isavuconazonium sulfate (Cayman Chemical, Ann Arbor, MI, USA) and 10 mM chlorhexidine were prepared in DMSO. Isavuconazonium sulfate was serially diluted two-fold to generate solutions ranging in concentration from 10 mM to 1.2 nM. Next, 0.5 μL of each of these isavuconazonium sulfate dilutions was added to 96-well white, flat bottom microplates (Greiner Bio-One, Kremsmünster, Austria). This was followed by the addition of 5 × 10^3^ trophozoites in 99.5 μL of PYG media to each well, giving final isavuconazonium sulfate concentrations ranging from 50 μM to 5.96 pM. Additionally, 0.5 μL of DMSO was added as a negative control (0.5% (*v*/*v*) DMSO), while 0.5 μL of chlorhexidine (50 μM) was added as a positive control. The plates were incubated for 48 h at 28 °C and 5% CO_2_. At 48 h, viability measurements were taken using the CellTiter-Glo luminescent cell viability assay (Promega, Madison, WI, USA) [7]. For the measurements, 25 μL of CellTiter-Glo was added to each well and shaken on an orbital shaker at 360 RPM for 10 min prior to luminescence readings on an EnVision 2104 Multilabel Reader (PerkinElmer, Waltham, MA, USA). Data from a minimum of three independent experiments (biological replicates) conducted in triplicate were analyzed on GraphPad Prism 6 to determine EC_50_ values.

### 4.3. Cyst Generation

Encystment of *Acanthamoeba* T4 strains was induced by culturing trophozoites in an encystation media (95 mM NaCl; 5 mM KCl; 8 mM MgSO_4_; 0.4 mM CaCl_2_; 1 mM NaHCO_3_; 20 mM Tris-HCl, pH 9.0) [19]. The trophozoite harvesting and encystation protocols were conducted as previously described by Shing et al. [7]. Briefly, trophozoites of *Acanthamoeba* T4 strains were centrifuged at 200× *g* for 5 min and washed in phosphate-buffered saline (PBS) three times prior to resuspension in encystation media. Then, 5 × 10^3^ cells in 99.5 µL were added to each well of a 96 well clear-bottom plate (Corning, Corning, NY, USA). The cells were incubated in encystation media to facilitate the encystation of trophozoites into cysts for 48 h prior to any cysticidal or combination experiments.

### 4.4. Determination of Cysticidal Activity

Cyst plate generation was conducted as previously described in Section 4.3 [7]. After 48 h, 0.5 μL of isavuconazonium sulfate solution was added to a final concentration of 200, 190, 180, 170, 160, 150, 140, 130, 120, 110, or 100 μM. To serve as negative and positive controls, 0.5% (*v*/*v*) DMSO and 461.85 μM PHMB were used, respectively. The cysts were incubated for 48 h. Afterwards, the wells were washed four times with 100 μL of PBS before the addition of 100 μL of PYG medium. The cysts were then incubated for one week and imaged by an ImageXpress Micro XLS (Molecular Devices, San Jose, CA, USA) at 200× magnification. The PYG growth media were exchanged for fresh media on day 3 and day 5. The images were manually reviewed for excysted trophozoites, and cysticidal activity was defined as having no trophozoites by day 7. Image brightness and contrast were adjusted by ImageJ. All experiments were conducted in triplicate and images were analyzed from a minimum of four independent experiments.

### 4.5. Effect of Combination of Isavuconazonium and PHMB on Cysts

Isavuconazonium was evaluated in combination with PHMB to assess potential synergy. After generating a plate of cysts using the methods described in Section 4.3, the cysts were treated with a checkerboard dilution scheme.

Isavuconazonium sulfate was serially diluted two-fold to generate solutions ranging in concentration from 65.7 mM to 512 μM. PHMB was serially diluted two-fold to generate solutions ranging in concentration from 64.7 mM to 504 μM. Then, 0.25 μL of each of the isavuconazonium sulfate and PHMB dilutions were added to each well of the plate to generate various concentration combinations. Isavuconazonium’s dilution gradient varied horizontally across the plate to give final concentrations ranging from 164.14 to 1.28 μM. PHMB’s dilution gradient varied vertically across the plate to give final concentrations ranging from 161.15 to 1.26 μM. As a negative control, 0.5% (*v*/*v*) DMSO was used, while 461.85 μM PHMB served as a positive control. Additionally, isavuconazonium sulfate and PHMB were also tested in monotherapy using the same final concentrations (164.14 to 1.28 μM and 161.15 to 1.26 μM, respectively) in separate columns.

After treatment for 48 h, the plate was washed with PBS and imaged for one week on the ImageXpress Micro XLS as described in Section 4.4. Images from three independent experiments were manually reviewed for excystation.

## 5. Conclusions

In this work, we tested isavuconazonium sulfate against *Acanthamoeba* T4 trophozoites and cysts to evaluate its potential as an anti-*Acanthamoeba* treatment. We found multiple T4 strains of *Acanthamoeba* to be susceptible to isavuconazonium, with appreciable activity against trophozoites even at low nanomolar concentrations. Cysts required significantly higher micromolar concentrations to prevent excystation. These findings suggest isavuconazonium to be potentially useful for clinical treatment of *Acanthamoeba* keratitis. Future studies should focus on in vivo animal models to validate isavuconazonium as a treatment option.

## Figures and Tables

**Table 1:** EC_50_ values of isavuconazonium sulfate against trophozoites of *Acanthamoeba* T4 strains.

Inhibitor	Strain	Mean (µM)	95% Lower CL (µM) ^a^	95% Upper CL (µM) ^a^
Isavuconazonium sulfate	Ma	0.001	0.001	0.002
	CDC:V240	0.037	0.027	0.049
	MEEI 0184	0.024	0.021	0.027
**Standards of care**				
Chlorhexidine [7]	Ma	1.7	1.4	1.9
	CDC:V240	1.1	1.0	1.2
	MEEI 0184	1	0.9	1.1
PHMB [7]	Ma	7.2	6.6	8.0
	CDC:V240	11.8	10.5	13.4
	MEEI 0184	4.6	3.0	7.1

^a^ CL, confidence limit.

## Data Availability

Data is contained within the article.
